# Predictive effect of triglyceride‑glucose index on clinical events in patients with type 2 diabetes mellitus and acute myocardial infarction: results from an observational cohort study in China

**DOI:** 10.1186/s12933-021-01236-3

**Published:** 2021-02-11

**Authors:** Yue Zhang, Xiaosong Ding, Bing Hua, Qingbo Liu, Hui Gao, Hui Chen, Xue-Qiao Zhao, Weiping Li, Hongwei Li

**Affiliations:** 1 Department of Cardiology, Cardiovascular Center, Beijing Friendship Hospital, Capital Medical University, 95 Yongan Road, Beijing, 100050 China; 2 Clinical Atherosclerosis Research Lab, Division of Cardiology, University of Washington, Seattle, WA USA; 3 Beijing Key Laboratory of Metabolic Disorder Related Cardiovascular Disease, Beijing, China; 4 Department of Geriatrics, Cardiovascular Center, Beijing Friendship Hospital, Capital Medical University, Beijing, China

**Keywords:** Insulin resistance (IR), Triglyceride-glucose index (TyG index), Type 2 diabetes mellitus (T_2_DM), Acute myocardial infarction (AMI), Major adverse cardiac and cerebral events (MACCEs)

## Abstract

**Background:**

Triglyceride glucose (TyG) index is considered a reliable alternative marker of insulin resistance and an independent predictor of cardiovascular (CV) outcomes. However, the prognostic value of TyG index in patients with type 2 diabetes mellitus (T_2_DM) and acute myocardial infarction (AMI) remains unclear.

**Methods:**

A total of 1932 consecutive patients with T_2_DM and AMI were enrolled in this study. Patients were divided into tertiles according to their TyG index levels. The incidence of major adverse cardiac and cerebral events (MACCEs) was recorded. The TyG index was calculated as the ln [fasting triglycerides (mg/dL) × fasting plasma glucose (mg/dL)/2].

**Results:**

Competing risk regression revealed that the TyG index was positively associated with CV death [2.71(1.92 to 3.83), *p* < 0.001], non-fatal MI [2.02(1.32 to 3.11), *p* = 0.001], cardiac rehospitalization [2.42(1.81 to 3.24), *p* < 0.001], revascularization [2.41(1.63 to 3.55), *p* < 0.001] and composite MACCEs [2.32(1.92 to 2.80), *p* < 0.001]. The area under ROC curve of the TyG index for predicting the occurrence of MACCEs was 0.604 [(0.578 to 0.630), *p* < 0.001], with the cut-off value of 9.30. The addition of TyG index to a baseline risk model had an incremental effect on the predictive value for MACCEs [net reclassification improvement (NRI): 0.190 (0.094 to 0.337); integrated discrimination improvement (IDI): 0.027 (0.013 to 0.041); C-index: 0.685 (0.663 to 0.707), all *p* < 0.001].

**Conclusions:**

The TyG index was significantly associated with MACCEs, suggesting that the TyG index may be a valid marker for risk stratification and prognosis in patients with T_2_DM and AMI.

*Trial registration* Retrospectively registered.

## Introduction

Acute myocardial infarction (AMI) has been recognized as the leading cause of morbidity and mortality of cardiovascular diseases(CVDs) worldwide [1]. The World Bank estimated that the number of individuals with MI in China will increase to 23 million by 2030 [2]. What’s more, some AMI patients remain at high risk for recurrent cardiovascular events (CVEs) despite the use of current guideline-recommended treatment. This risk is particularly high among patients with type 2 diabetes mellitus (T_2_DM), accounting for approximately 37% of AMI cases in China, and is classified as extreme-risk group for recurrent CVEs [3]. Studies have shown that T_2_DM is significantly correlated with more complex coronary lesions and worse prognosis in AMI patients [4, 5].Therefore, early identification of the residual risk factors of AMI patients with T_2_DM is crucial for better clinical management to reduce future CVEs.

Insulin resistance (IR), a crucial mediator of metabolic disorders, not only contributes to the pathogenesis of CVDs, but also correlates with adverse cardiovascular (CV) outcomes [6–8]. Although the hyperinsulinemic-euglycemic clamp is the gold-standard test for IR assessment [9], it is not commonly used in clinical settings and large population studies due to the complex testing process [10]. Given that IR is significantly associated with the chronic increase in plasma glucose and triglycerides (TGs) [11], researchers hypothesized that the combination of plasma glucose and TGs might predict IR. Triglyceride glucose (TyG) index, which combines fasting plasma glucose (FPG) and TGs levels, has been shown to be significantly correlated with IR measured by the hyperinsulinaemic-euglycaemic clamp test [12] and homeostasis model assessment of IR (HOMA-IR) [13]. The TyG index was regarded as a reproducible, reliable, and valid surrogate marker of IR [12, 14, 15]. Numerous studies have indicated that the TyG index was significantly correlated with the occurrence of CVDs and poor CV prognosis [16–25]. However, no previous study has exclusively investigated the predictive value of the TyG index for adverse CVEs in AMI patients with T_2_DM. Our study was to fill this knowledge gap.

## Methods

### Study population

Study subjects were identified from the Cardiovascular Center of Beijing Friendship Hospital Database (CBD) Bank. A total of 5169 consecutive patients were diagnosed with AMI and underwent coronary angiography from January 2013 to August 2020. Of the 5169 patients, 3237 were excluded according to the exclusion criteria, which were (1) without T_2_DM, (2) with estimated glomerular filtration rate (eGFR) < 30 mL/min/1.73m^2^ or chronic dialysis, severe hepatic dysfunction, severe acute infection, malignant tumor, suspected familial hypertriglyceridemia [plasma TGs ≥ 5.65 mmol/L], (3) with cardiogenic shock, prior coronary artery bypass graft (CABG), severe valvulopathy or congenital heart disease requiring cardiac surgery, (4) lack of clinical or follow-up data. Finally, 1932 patients were included in this analysis. The patients were divided into 2 groups according to the occurrence of MACCEs during the follow-up: the MACCEs group (n = 735) and the No-MACCEs group (n = 1197). In addition, the patients were also divided into tertiles according to their TyG index levels (TyG index ≤ 8.91 group, n = 647; 8.91 < TyG index < 9.54 group, n = 639; TyG index ≥ 9.54 group, n = 646). All patients were followed up till October 31, 2020 with a median follow up of 26.8 (IQR: 12.4, 50.7) months.

### Data collections and definitions

The data collection process was approved by the Institutional Review Board of Beijing Friendship Hospital affiliated to Capital Medical University and was in accordance with the Declaration of Helsinki.

Patients’ demographics, medical history, laboratory test results, echocardiographic, and angiographic evaluation results were collected and verified using an electronic medical recording system. The concentrations of TGs and FPG in the first fasting blood samples during the stay in the hospital, which were obtained after at least 10 h of fasting, were determined at the central laboratory of Beijing Friendship Hospital. The TyG index was calculated as ln [fasting TGs (mg/dL) × FPG (mg/dL)/2] [12]. The Single Point Insulin Sensitivity Estimator(SPISE) and TGs/high-density lipoprotein cholesterol(HDL-C) ratio have been proven to be effective surrogate indexs for insulin sensitivity, so they are also included in the baseline clinical characteristics. The novel formula for SPISE was computed as follows: SPISE = 600 × HDL-C^0.185^/(TGs^0.2^ × body mass index(BMI)^1.338^) [26], with fasting HDL-C (mg/dL), fasting TGs (mg/dL), and BMI (kg/m^2^). The TGs/HDL-C ratio was calculated as TGs (mg/dL)/HDL-C(mg/dL). The outcomes from major adverse cardiac and cerebral events(MACCEs) were collected and recorded during clinical follow-up visits.

Criteria for T_2_DM include: (1) previously diagnosed T_2_DM under treatment of antidiabetic medication; (2) the typical symptoms of DM with a FPG ≥ 7.0 mmol/L, and/or random blood glucose (RBG) ≥ 11.1 mmol/L, and/or 2-h plasma glucose level after oral glucose tolerance test (OGTT) ≥ 11.1 mmol/L [27]. Hypertension (HT) is defined as the blood pressure is greater than or equal to 140/90 mmHg three times on different days, or the antihypertensive drugs are used. The criteria for dyslipidaemia is that fasting total cholesterol(TC) > 200 mg/dL, low-density lipoprotein cholesterol(LDL-C) > 130 mg/dL, TGs > 150 mg/dL, HDL-C < 40 mg/dL, or previous use of lipid-lowering drugs. AMI, including non-ST-segment elevation myocardial infarction(NSTEMI) and ST-elevation myocardial infarction(STEMI), was defined as chest pain with new ST-segment changes and elevation of myocardial necrosis markers to at least twice of the upper limit of the normal range. MACCEs included all-cause death, non-fatal MI, non-fatal stroke, cardiac rehospitalization (admission because of angina or heart failure), and revascularization. CV death was defined as fatal stroke and MI, sudden death, and other cardiac death. All-cause death was defined as the incidence of CV death or non-CV death. Non-fatal stroke, including ischemic and hemorrhagic stroke, was defined as cerebral dysfunction caused by cerebral vascular obstruction or sudden rupture and was diagnosed based on signs of neurological dysfunction or evidence of brain imaging. Cardiac rehospitalization refers to rehospitalization for angina pectoris or heart failure. Any coronary revascularization was defined as a revascularization of the target vessel or non-target vessels.

### Statistical analyses

Continuous variables were presented as mean ± standard deviation (SD) or median (IQR). Comparisons between the 2 study groups were analyzed by Student’s t-test or Mann–Whitney U-test. Categorical variables were expressed as number and percentage and compared using the Pearson chi-square test or Fisher’s exact test. Two -sample T-test power analysis showed that the testing power of TyG index is 1. Baseline variables that were significantly correlated with MACCEs by univariate analysis and clinically relevant were entered into the multivariate model. Also, intercorrelations among variables were taken into consideration in the multivariate analysis. Considering that there is a competitive risk relationship between all-cause death and other events, the competing risk model is used to verify the independent predictive effect of the TyG index on each type of MACCEs. The cumulative incidence of MACCEs was estimated by competing risk regression curves. Receiver-operating characteristic (ROC) curve analysis was performed to determine the optimal cutoff point value of TyG index for predicting MACCEs. We also calculated net reclassification improvement (NRI) and integrated discrimination improvement (IDI) to determine the extent to which the addition of TyG index improves the predictive power of existing baseline risk model. Statistical tests were performed with IBM SPSS statistics 24, StataMP 14 and the R Programming Language. A two-tailed *p* value < 0.05 was regarded as statistically significant.

## Results

### Baseline characteristics of patients

Baseline characteristics of the total population and groups stratified by with or without MACCEs were presented in Table 1. The TyG index level and the proportion of the patients with TyG ≥ 9.54 were significantly higher in MACCEs group than those in the No-MACCEs group. Patients with MACCEs showed higher age, TGs/HDL-C ratio, hypersensitive C-reactive protein (hs-CRP), FPG, TGs and creatinine, longer duration of diabetes, and higher prevalence of previous stroke and percutaneous coronary intervention (PCI) history. In addition, patients in MACCEs group had lower levels of hemoglobin, albumin and left ventricular ejection fraction (LVEF). As for the angiographic findings, those with MACCEs showed lower proportions of left main coronary artery (LM)/three-vessel, proximal left anterior descending (LAD) and PCI/CABG treatment during hospitalization.

**Table 1:** Baseline clinical characteristics of the patients stratified by MACCEs

Variable	Total population n = 1932	No-MACCEs n = 1197	MACCEs n = 735	*p* value
Insulin sensitivity surrogate index				
TyG index	9.26 ± 0.73	9.17 ± 0.72	9.42 ± 0.72	< 0.001
TyG ≤ 8.91	647 (33.5)	467(39.0)	180 (24.5)	< 0.001
8.91 < TyG < 9.54	639 (33.1)	401(33.5)	238 (32.4)	
TyG ≥ 9.54	646 (33.4)	329(27.5)	317 (43.1)	
TGs/HDL-C ratio	3.6 (2.4,5.6)	3.3(2.3,5.1)	4.2 (2.7,6.3)	< 0.001
SPISE index	5.9 ± 1.5	5.9 ± 1.5	5.8 ± 1.5	0.099
Age, years	65.4 ± 12.0	64.1 ± 11.6	67.4 ± 12.4	< 0.001
Male gender	1324 (68.5)	825(68.9)	499 (67.9)	0.636
BMI, kg/m^2^	25.8 ± 3.5	25.9 ± 3.5	25.6 ± 3.7	0.150
SBP, mmHg	130.9 ± 22.2	130.3 ± 21.4	131.7 ± 23.5	0.187
DBP, mmHg	73.8 ± 12.6	73.9 ± 12.3	73.7 ± 13.0	0.640
Medical history				
Current/ex-Smoker	1102 (57.0)	698 (58.3)	404 (55.0)	0.149
Duration of diabetes, years	6.0 (1.0,12.0)	6.0 (1.0,10.0)	8.0 (1.0,14.0)	< 0.001
CKD	113 (5.8)	51 (4.3)	62 (8.4)	< 0.001
Stroke	396 (20.5)	206 (17.2)	190 (25.9)	< 0.001
Hypertension	1450 (75.1)	887 (74.1)	563 (76.6)	0.218
Dyslipidemia	931 (48.2)	584 (48.8)	347 (47.2)	0.500
Previous MI	218 (11.3)	135 (11.3)	83 (11.3)	0.992
Past PCI	321 (16.6)	183 (15.3)	138 (18.8)	0.046
Medication used before admission				
Antiplatelet agent	618 (32.0)	364 (30.4)	254 (34.6)	0.058
ACEI/ARB	590(30.5)	364(30.4)	226(30.7)	0.875
Beta-blocker	291(15.1)	171(14.3)	120(16.3)	0.223
Statins	404(20.9)	249(20.8)	155(21.1)	0.881
Laboratory values				
WBC, 10^9^/L	8.1 (6.4,10.1)	8.0 (6.5,9.9)	8.2 (6.4,10.3)	0.342
Hemoglobin, g/L	132.3 ± 20.0	133.9 ± 19.2	129.8 ± 20.9	< 0.001
Hs-CRP, mg/L	12.2 (5.4,12.5)	12.1 (6.3,12.5)	12.3 (4.7,15.0)	0.006
RBG at admission, mmol/L	11.1 (8.3,14.3)	10.8 (8.1,14.0)	11.5 (8.5,14.6)	0.026
FPG, mmol/L	8.0 (6.3,10.4)	7.9 (6.2,10.1)	8.3 (6.4,11.1)	0.001
HbA1c,%	7.8 ± 1.7	7.8 ± 1.7	7.8 ± 1.6	0.220
Albumin, g/L	36.0 ± 3.2	36.2 ± 3.0	35.8 ± 3.5	0.015
Creatinine, umol/L	80.9 (68.4,95.5)	77.5 (66.2,92.2)	85.9 (72.3,106.0)	< 0.001
eGFR, ml/min/1.73m^2^	82.1 (63.2,98.5)	86.9 (68.8,101.8)	74.2 (56.1,90.9)	< 0.001
TC, mmol/L	4.38 (3.68,5.04)	4.41(3.69,5.10)	4.35 (3.65,5.00)	0.276
TGs, mmol/L	1.51 (1.07,2.26)	1.40 (1.01,2.09)	1.78 (1.24,2.55)	< 0.001
LDL-C, mmol/L	2.58 ± 0.79	2.60 ± 0.79	2.56 ± 0.79	0.315
HDL-C, mmol/L	1.01 ± 0.24	1.00 ± 0.24	1.02 ± 0.25	0.253
Initial diagnosis				
NSTEMI	1042 (53.9)	630 (52.6)	412 (56.1)	0.143
STEMI	890 (46.1)	567 (47.4)	323 (43.9)	
Echocardiography				
LVEF	57.4 ± 10.3	58.8 ± 9.4	55.0 ± 11.3	< 0.001
Angiography findings				
LM/three-vessel	1369 (70.9)	881 (73.6)	488 (66.4)	0.001
Proximal LAD	948 (49.1)	631 (52.7)	317 (43.1)	< 0.001
In-hospital treatment				
PCI/CABG	1501 (77.7)	1003 (83.8)	498 (67.8)	< 0.001
Antiplatelet agent	1854 (96.0)	1157 (96.7)	697 (94.8)	0.047
ACEI/ARB	1291 (66.8)	816 (68.2)	475 (64.6)	0.108
Beta-blocker	1434 (74.2)	905 (75.6)	529 (72.0)	0.076
Statins	1673 (86.6)	1072 (89.6)	601 (81.8)	< 0.001
Hypoglycemic agents				
Metformin	655 (33.9)	437 (36.5)	218 (29.7)	0.002
Alpha-glucosidase inhibitor	1209 (62.6)	760 (63.5)	449 (61.1)	0.289
Sulfonylurea	434 (22.5)	281 (23.5)	153 (20.8)	0.174
DPP-4i	20 (1.0)	16 (1.3)	4 (0.5)	0.095
Insulin	577 (29.9)	312 (26.1)	265 (36.1)	< 0.001

Dates are presented as mean ± SD, median (IQR) or number (%)

MACCEs, major adverse cardiac and cerebral events; TyG, triglyceride-glucose index; TGs, triglycerides; HDL-C, high-density lipoprotein cholesterol; SPISE index, the Single Point Insulin Sensitivity Estimator; BMI, body mass index; SBP, systolic blood pressure; DBP, diastolic blood pressure; CKD, chronic kidney disease; MI, myocardial infarction; PCI, percutaneous coronary intervention; ACEI/ARB, angiotensin-converting enzyme inhibitor/angiotensin receptor blocker; WBC, white blood cell; Hs-CRP, hypersensitive C-reactive protein; RBG, random blood glucose; FPG, fasting plasma glucose; HbA1c, glycated hemoglobin; eGFR, estimated glomerular filtration rate; TC, total cholesterol; LDL-C, low-density lipoprotein cholesterol; NSTEMI, Non-ST-segment elevation myocardial infarction; STEMI, ST-elevation myocardial infarction; LVEF, left ventricular ejection fraction; LM, left main coronary artery; LAD, left anterior descending; CABG, coronary artery bypass graft; DPP-4i, dipeptidyl peptidase-4 inhibitor

### TyG index predicted the occurrence of MACCEs

Univariate and multivariate Cox proportional hazards regression analyses and predictors for composite MACCEs were presented in Table 2. In the univariate analysis, the predictor associated with MACCEs occurrence were TyG index, age, BMI, duration of diabetes, chronic kidney disease (CKD), previous stroke, past PCI, antiplatelet agent used before admission, white blood cell(WBC), hs-CRP, hemoglobin, FPG, RBG at admission, albumin, creatinine, eGFR, TGs, LVEF, LM/three-disease, proximal LAD, in-hospital treatment[PCI/CABG, antiplatelet agent, angiotensin-converting enzyme inhibitor/angiotensin receptor blocker (ACEI/ARB), beta-blocker and statins] and hypoglycemic agents(insulin). FPG, RBG at admission, TGs and TyG index had a high correlation (*p* < 0.001). In addition, CKD and creatinine were significantly correlated with eGFR (*p* < 0.001), and hs-CRP was significantly correlated with WBC (*p* < 0.001). Therefore, FPG, RBG at admission, TGs, CKD, creatinine and hs-CRP were not included in the multivariate model. After adjusting for age, BMI and other confounding factors, multivariate Cox proportional hazards regression analysis showed that TyG index, age, previous stroke, WBC, eGFR, LVEF and in-hospital treatment(PCI/CABG, antiplatelet agent, beta-blocker and statins) independently predicted the occurrence of MACCEs in patients with AMI and T_2_DM.

**Table 2:** Independent predictors of composite MACCEs

	Univariate		Multivariate	
	HR (95%CI)	*p* value	Adjusted HR (95%CI)	*p* value
Insulin sensitivity surrogate index				
TyG index				
TyG ≤ 8.91	Reference		Reference	
8.91 < TyG < 9.54	1.33 (1.09,1.61)	0.004	1.58(1.33,1.88)	< 0.001
TyG ≥ 9.54	1.93 (1.61,2.32)	< 0.001	2.32 (1.92,2.80)	< 0.001
TGs/HDL-C ratio	1.01 (0.99,1.03)	0.675		
SPISE index	0.98 (0.93,1.03)	0.318		
Age, y	1.03 (1.01,1.04)	< 0.001	1.02 (1.01,1.03)	< 0.001
Male gender	1.04 (0.88.1.20)	0.642		
BMI, kg/m^2^	1.02 (1.01,1.04)	0.046	0.99 (0.97,1.01)	0.357
SBP, mmHg	1.01 (0.99,1.02)	0.546		
DBP, mmHg	0.99 (0.98,1.00)	0.461		
Medical history				
Current/ex-Smoker	0.93 (0.80,1.07)	0.302		
Duration of diabetes, years	1.02 (1.01,1.03)	< 0.001	1.02 (0.99,1.03)	0.857
CKD	1.85 (1.43,2.40)	< 0.001		
Stroke	1.59 (1.35,1.87)	< 0.001	1.38 (1.16,1.63)	< 0.001
Hypertension	1.17 (0.98,1.39)	0.075		
Dyslipidemia	1.04 (0.90,1.21)	0.558		
Previous MI	1.15 (0.91,1.44)	0.243		
Past PCI	1.25 (1.04,1.50)	0.020	0.87 (0.71,1.08)	0.205
Medication used before admission				
Antiplatelet agent	1.25 (1.08,1.46)	0.004	0.95 (0.80,1.13)	0.542
ACEI/ARB	1.09 (0.94,1.28)	0.261		
Beta-blocker	1.20 (0.98,1.45)	0.074		
Statins	1.15(0.96,1.38)	0.120		
Laboratory values				
WBC,10^9^/L	1.04 (1.02,1.05)	< 0.001	1.03 (1.02,1.04)	< 0.001
Hemoglobin, g/L	0.98 (0.97,0.99)	< 0.001	0.99 (0.98,1.01)	0.776
Hs-CRP, mg/L	1.02 (1.01,1.03)	< 0.001		
RBG at admission, mmol/L	1.02 (1.01,1.03)	0.042		
FPG, mmol/L	1.05 (1.03, 1.08)	< 0.001		
HbA1c, %	1.04 (0.99,1.09)	0.066		
Albumin, g/L	0.97 (0.95,0.99)	0.004	1.01 (0.98,1.03)	0.826
Creatinine, umol/L	1.02 (1.01,1.03)	< 0.001		
eGFR, ml/min/1.73m^2^	0.98 (0.96,0.99)	< 0.001	0.98(0.97,0.99)	0.004
TC, mmol/L	1.01 (0.95,1.09)	0.673		
TGs, mmol/L	1.08 (1.01,1.12)	< 0.001		
LDL-C, mmol/L	1.01 (0.92,1.11)	0.821		
HDL-C, mmol/L	0.94 (0.70,1.27)	0.705		
Echocardiography				
LVEF,%	0.97 (0.96,0.98)	< 0.001	0.98 (0.97,0.99)	< 0.001
Angiography findings				
LM/three-disease	1.39 (1.19,1.62)	< 0.001	1.15 (0.92,1.42)	0.222
Proximal LAD	1.38 (1.20,1.60)	< 0.001	1.13 (0.96,1.33)	0.150
In-hospital treatment				
PCI/CABG	0.52 (0.45,0.61)	< 0.001	0.63 (0.53,0.74)	< 0.001
Antiplatelet agent	0.67 (0.49,0.94)	0.018	0.61 (0.42,0.88)	0.008
ACEI/ARB	0.77 (0.66,0.89)	0.001	0.89 (0.76,1.05)	0.167
Beta-blocker	0.75 (0.64,0.88)	< 0.001	0.79 (0.67,0.94)	0.009
Statins	0.56 (0.47,0.68)	< 0.001	0.64 (0.52,0.78)	< 0.001
Hypoglycemic agents				
Metformin	0.86 (0.74,1.01)	0.068	0.98 (0.83,1.16)	0.804
Alpha-glucosidase inhibitor	0.94 (0.81,1.08)	0.367		
Sulfonylurea	0.85 (0.71,1.02)	0.074	0.88(0.73,1.06)	0.174
DPP-4i	0.78 (0.29,2.08)	0.619		
Insulin	1.40 (1.20,1.63)	< 0.001	1.13(0.96,1.33)	0.139

MACCEs, major adverse cardiac and cerebral events; TyG, triglyceride-glucose index; TGs, triglycerides; HDL-C, high-density lipoprotein cholesterol; SPISE index, the Single Point Insulin Sensitivity Estimator; BMI, body mass index; SBP, systolic blood pressure; DBP, diastolic blood pressure; CKD, chronic kidney disease; MI, myocardial infarction; PCI, percutaneous coronary intervention; ACEI/ARB, angiotensin-converting enzyme inhibitor/angiotensin receptor blocker; WBC, white blood cell; Hs-CRP, hypersensitive C-reactive protein; RBG, random blood glucose; FPG, fasting plasma glucose; HbA1c, glycated hemoglobin; eGFR, estimated glomerular filtration rate; TC, total cholesterol; LDL-C, low-density lipoprotein cholesterol; LVEF, left ventricular ejection fraction; LM, left main coronary artery; LAD, left anterior descending; CABG, coronary artery bypass graft; DPP-4i, dipeptidyl peptidase-4 inhibitor

**Fig. 1:**
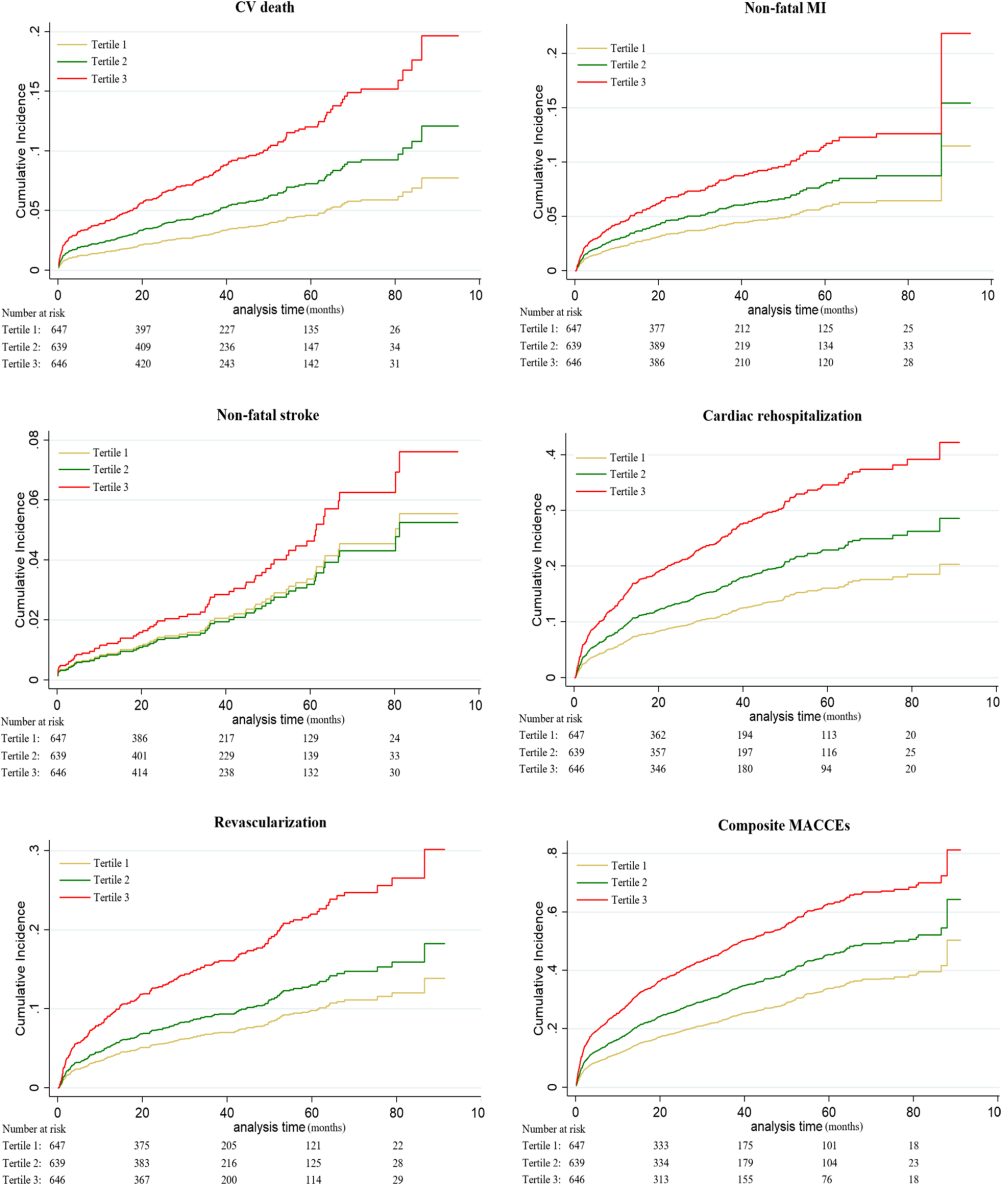
Competing risk regression curves for CV death, non-fatal MI, non-fatal stroke, cardiac rehospitalization, revascularization and composite MACCEs of the TyG ≤ 8.91 group (Tertile 1, yellow line), the 8.91 < TyG < 9.54 group (Tertile 2, green line) and the TyG ≥ 9.54 group (Tertile 3, red line). TyG, triglyceride-glucose index; CV, cardiovascular; MI, myocardial infarction; MACCEs, major adverse cardiac and cerebral events

During the median of 26.8-month follow-up, MACCEs occurred in 735 (38.0%) patients [all-cause death: 292 (15.1%); CV death: 233 (12.1%); non-fatal MI: 161 (8.3%); non-fatal stroke: 76 (3.9%); cardiac rehospitalization: 354 (18.3%); revascularization: 226 (11.7%)]. Figure 1 and Table 3 showed the competing risk regression analysis for MACCEs. On unadjusted competing risk modeling, the cumulative incidence of CV death, non-fatal MI, cardiac rehospitalization, revascularization and composite MACCEs rose significantly with elevated TyG index levels (all *p* < 0.05). Notably, after adjusting for age, BMI and other potential confounding factors, multivariate-adjusted hazard ratio (HR) also increased with rising TyG index levels for all-cause death, CV death, non-fatal MI, cardiac rehospitalization, revascularization and composite MACCEs (all *p* < 0.05).

**Table 3:** Competing risk model of MACCEs

	% (Events)	Unadjusted HR (95% CI)	*p* value	Adjusted HR (95% CI)	*p* value
All cause death					
TyG ≤ 8.91	12.8% (83)	Ref	-/-	Ref	-/-
8.91 < TyG < 9.54	15.8% (101)	1.18 (0.86,1.58)	0.255	1.67 (1.24,2.25)	0.001
TyG ≥ 9.54	16.7% (108)	1.26 (0.95,1.68)	0.112	2.35 (1.72,3.20)	< 0.001
CV death					
TyG ≤ 8.91	9.3% (60)	Ref	-/-	Ref	-/-
8.91 < TyG < 9.54	11.9% (76)	1.24 (0.88,1.73)	0.217	1.60 (1.11,2.30)	0.012
TyG ≥ 9.54	15.0% (97)	1.60 (1.16,2.20)	0.004	2.71 (1.92,3.83)	< 0.001
Non-fatal MI					
TyG ≤ 8.91	5.9% (38)	Ref	-/-	Ref	-/-
8.91 < TyG < 9.54	8.0% (51)	1.29 (0.85,1.96)	0.214	1.37 (0.90,2.10)	0.143
TyG ≥ 9.54	11.1% (72)	1.93 (1.28,1.90)	0.001	2.02 (1.32,3.11)	0.001
Non-fatal stroke					
TyG ≤ 8.91	3.9% (25)	Ref	-/-	Ref	-/-
8.91 < TyG < 9.54	3.4% (22)	0.94 (0.52,1.69)	0.830	0.94 (0.50,1.78)	0.859
TyG ≥ 9.54	4.5% (29)	1.20 (0.69,2.08)	0.529	1.39 (0.73,2.63)	0.315
Cardiac rehospitalization					
TyG ≤ 8.91	11.3% (73)	Ref	-/-	Ref	-/-
8.91 < TyG < 9.54	17.4% (111)	1.51 (1.13,2.03)	0.006	1.48 (1.10,2.01)	0.011
TyG ≥ 9.54	26.3% (170)	2.46 (1.87,3.24)	< 0.001	2.42 (1.81,3.24)	< 0.001
Revascularization					
TyG ≤ 8.91	6.5% (42)	Ref	-/-	Ref	-/-
8.91 < TyG < 9.54	10.6% (68)	1.58 (1.08,2.31)	0.020	1.35 (0.90,2.02)	0.142
TyG ≥ 9.54	18.0% (116)	2.83 (1.99,4.04)	< 0.001	2.41 (1.63,3.55)	< 0.001
Composite MACCEs					
TyG ≤ 8.91	27.8% (180)	Ref	-/-	Ref	-/-
8.91 < TyG < 9.54	37.2% (238)	1.34 (1.10,1.64)	0.004	1.58 (1.33,1.88)	< 0.001
TyG ≥ 9.54	49.1% (317)	2.03 (1.68,2.45)	< 0.001	2.32 (1.92,2.80)	< 0.001

Adjusted factors included TyG index, age, BMI, history of stroke and PCI, antiplatelet agent used before admission, WBC, hemoglobin, albumin, eGFR, LVEF, angiography findings (LM/three-disease and proximal LAD), in-hospital treatment(PCI/CABG, antiplatelet agent, ACEI/ARB, beta-blocker, statins) and hypoglycemic agents(Metformin, Alpha-glucosidase inhibitor, DPP-4i and insulin)

MACCEs, major adverse cardiac and cerebral events; TyG, triglyceride-glucose index; CV, cardiovascular; MI, myocardial infarction; BMI, body mass index; PCI, percutaneous coronary intervention; WBC,white blood cell; eGFR, estimated glomerular filtration rate; LVEF, left ventricular ejection fraction; LM, left main coronary artery; LAD, left anterior descending; CABG, coronary artery bypass graft; ACEI/ARB, angiotensin-converting enzyme inhibitor/angiotensin receptor blocker; DPP-4i, dipeptidyl peptidase-4 inhibitor; HR, hazard ratio; CI, confidence interval; Ref, reference

### ROC curve analysis of the value of TyG index

**Fig. 2:**
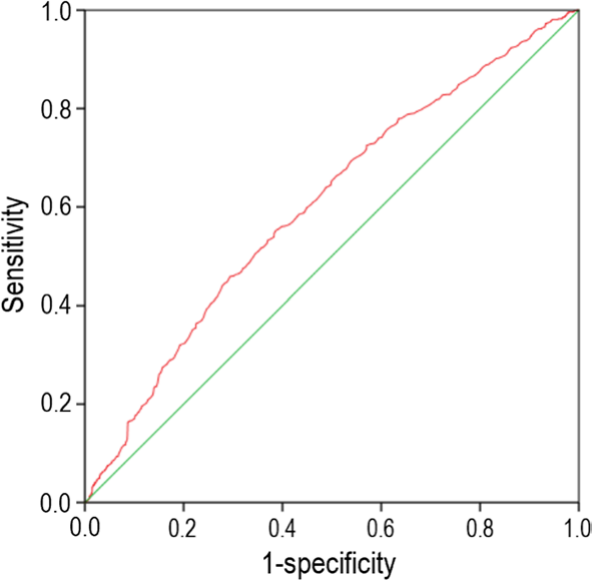
The receiver operating characteristic (ROC) curves of the TyG index as a marker to predict composite MACCEs in patients with T_2_DM and AMI. The area under ROC curves (AUCs) of the TyG index for predicting the occurrence of MACCEs was 0.604 (95% CI 0.578–0.630; *p* < 0.001). The cut-off value of TyG index to predict MACCEs was 9.30, the sensitivity was 0.552, and the specificity was 0.613

The area under ROC curves (AUCs) of the TyG index for predicting the occurrence of MACCEs was 0.604 (95% CI 0.578–0.630; *p* < 0.001) (Fig. 2). The cut-off value of TyG index to predict MACCEs was 9.30, the sensitivity was 0.552, and the specificity was 0.613.

### Incremental effect of TyG index on predictive value for MACCEs

Table 4 showed that compared with the glycated hemoglobin (HbA1c), FPG and TGs, the addition of TyG index significantly improved the reclassification and discrimination ability beyond the baseline risk model with NRI of 0.190 and IDI of 0.027 (both *p* < 0.001). In addition, the C-index of the baseline risk model [0.659 (0.638 to 0.681), *p* < 0.001] changed after addition of the TyG-index [0.685(0.663 to 0.707), *p* < 0.001].

**Table 4:** Evaluate the incremental predictive value and predictive power of various models with NRI, IDI and C-index

	Category-free NRI			IDI			C-index		
	Index	95% CI	*p* value	Index	95% CI	*p* value	Index	95% CI	*p* value
Baseline risk model			Ref			Ref	0.659	0.638 to 0.681	< 0.001
+ HbA1c	0.032	−0.063 to 0.094	0.228	0.005	0.000 to 0.013	0.084	0.661	0.638 to 0.683	< 0.001
+ FPG	0.095	0.015 to 0.150	0.020	0.007	0.001 to 0.015	0.016	0.664	0.641 to 0.686	< 0.001
+ TGs	0.111	0.030 to 0.164	0.020	0.010	0.007 to 0.020	0.012	0.676	0.654 to 0.697	< 0.001
+ TyG index	0.190	0.094 to 0.337	< 0.001	0.027	0.013 to 0.041	< 0.001	0.685	0.663 to 0.707	< 0.001

Baseline risk model including age, history of stroke, beta-blocker used before admission, WBC, eGFR, LVEF, in-hospital treatment(PCI/CABG, antiplatelet agent, beta-blocker and statins) and hypoglycemic agents( insulin)

NRI, net reclassification improvement; IDI, integrated discrimination improvement; HbA1c, glycated hemoglobin; FPG, fasting plasma glucose; TGs, triglycerides; TyG, triglyceride-glucose index; WBC, white blood cell; eGFR, estimated glomerular filtration rate; LVEF, left ventricular ejection fraction; PCI, percutaneous coronary intervention; CABG, coronary artery bypass graft; Ref, reference

### Independent association of TyG index with MACCEs in different subgroups

**Fig. 3:**
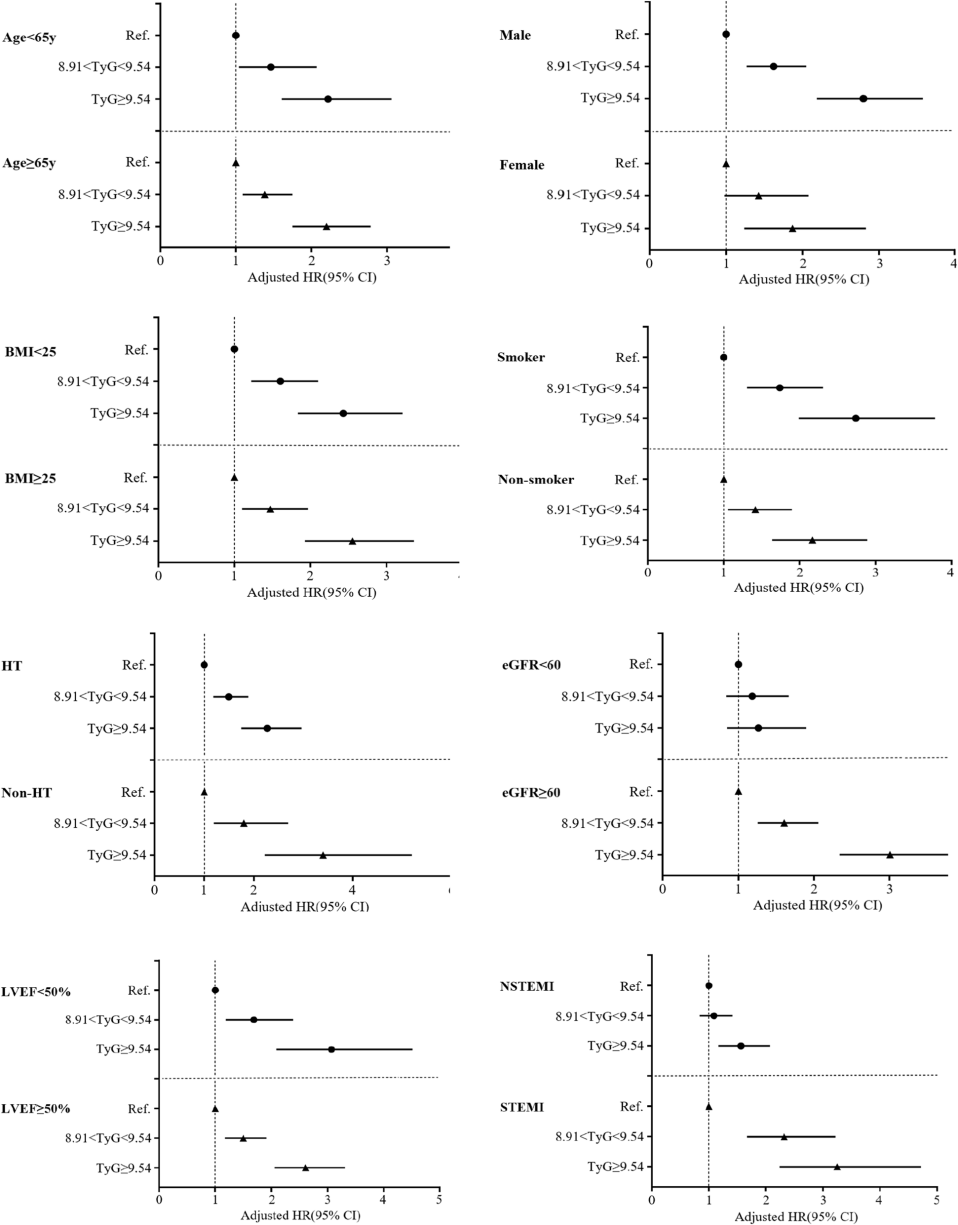
Forest plot of composite MACCEs according to different subgroups. Adjusted model included age, gender, BMI, SBP, DBP, previous MI, past PCI, history of stroke, current/ex-smoker, WBC, eGFR, albumin, TC, TGs, HDL-C, LDL-C, LVEF, hemoglobin, medication used before admission(antiplatelet agent, ACEI/ARB, beta-blocker and statins), in-hospital treatment(PCI/CABG, antiplatelet agent, ACEI/ARB, beta-blocker and statins) and hypoglycemic agents(metformin, alpha-glucosidase inhibitor, DPP-4i and insulin). MACCE, major adverse cardiac and cerebral events; TyG, triglyceride-glucose index; BMI, body mass index; HT, hypertension; eGFR, estimated glomerular filtration rate; LVEF, left ventricular ejection fraction; NSTEMI, Non-ST-segment elevation myocardial infarction; STEMI, ST-elevation myocardial infarction; SBP, systolic blood pressure; DBP, diastolic blood pressure; MI, myocardial infarction; PCI, percutaneous coronary intervention; WBC, white blood cell; TC, total cholesterol; TGs, triglycerides; LDL-C, low-density lipoprotein cholesterol; HDL-C, high-density lipoprotein cholesterol; ACEI/ARB, angiotensin-converting enzyme inhibitor/angiotensin receptor blocker; CABG, coronary artery bypass graft; DPP-4i, dipeptidyl peptidase-4 inhibitor; HR, hazard ratio; CI, confidence interval; Ref., reference(TyG ≤ 8.91 group)

Subgroup analysis was performed according to age, sex, BMI, smoker, HT, eGFR, LVEF and AMI type (Fig. 3). We found that the predictive effect of TyG index on MACCEs is effective in most subgroups, except for patients with eGFR < 60 ml/min/1.73m^2^.

## Discussion

To the best of our knowledge, this is the first study to explore the association between the TyG index and MACCEs in AMI patients with T_2_DM. Our main findings include: (1) the incidences of MACCEs significantly increased with the increase of TyG index, and (2) the TyG index was an independent predictor of MACCEs(all-cause death, CV death, non-fatal MI, cardiac rehospitalization, revascularization and composite MACCEs, and (3) The AUC of the TyG index for predicting the occurrence of MACCEs was 0.604 with a cut-off value of 9.30, and (4) The addition of TyG index to a baseline risk model had an incremental effect on the predictive value for MACCEs, and (5) the predictive effect of TyG index on MACCEs is ineffective in patients with eGFR < 60 ml/min/1.73 m^2^. According to this study, we confirmed that the TyG index was positively associated with increased MACCEs. Most importantly, this study suggested that a simple method of estimating IR may optimize the risk stratification of recurrent cardiovascular risk in AMI patients with T_2_DM.

IR is defined as a decrease in the efficiency of insulin in promoting glucose uptake and utilization, which is an indicator of abnormal metabolism. IR promotes the progression of CVDs by inducing glucose metabolism imbalance, altering systemic lipid metabolism, and causing endothelial dysfunction [11]. Several clinical studies found that IR was an important risk factor for CVDs and poor clinical outcomes [6, 28–30]. At present, the traditional methods of IR detection mainly include the hyperinsulinemic-euglycemic clamp and the HOMA-IR. However, due to the complexity and high cost of the detection process, the above two methods cannot be applied to clinical practice on a large scale. In order to solve this clinical problem, researchers have done a lot of studies on TyG index and found that it was a reliable surrogate marker of IR [12, 15]. Therefore, when the hyperinsulinaemic-euglycaemic clamp test and HOMA-IR cannot be measured, the TyG index be used to identify IR in clinical practice.

Researchers have done a lot of works to prove the predictive effect of TyG index on CVDs. Sánchez-Íñigo et al. suggested that a higher level of TyG index was significantly associated with an increased risk of developing CVDs independent of confounding factors, and the TyG index might be used to early identify the high-risk CVEs in healthy individuals [31]. Da Silva et al. demonstrated that the TyG index was positively associated with a higher prevalence of symptomatic coronary artery disease(CAD) in patients underwent secondary care for CVD [20]. Mao et al. firstly confirmed that the TyG index was positively associated with SYNTAX score and major adverse cardiovascular events (MACEs) in non-ST-segment elevation acute coronary syndrome (NSTE-ACS) population [22]. Additionally, a cohort study including 1092 STEMI patients who underwent PCI indicated that the incidences of composite MACCEs and all-cause death within 30 days, 6 months and 1 year were higher among those with highest level of TyG index (TyG index ≥ 9.608), and that the TyG index ≥ 9.608 was independently associated with an increased risk of MACCEs within 1 year [HR(95% CI) 1.53 (1.0 1, 2.06), *p* = 0 003] [21]. Considering that nearly one-third of ACS patients are combined with T_2_DM, and these patients are characterized by more complex coronary lesions, higher incidence of recurrent CVEs, and worse prognosis. Relevant studies about the TyG index in predicting CVEs in patients with ACS complicated with T_2_DM have been published in succession. Wang et al. followed up 2,531 ACS patients with T_2_DM for 3 years and found that the incidence of MACEs increased with the increase of TyG index, the TyG index was an independent predictor of MACEs, and the optimal TyG index cut-off for predicting MACEs was 9.323 [24]. A study by Ma et al. of 776 patients with T_2_DM and ACS who underwent PCI also showed that the TyG index was significantly associated with adverse CV outcomes, including all-cause mortality, non-fatal stroke, non-fatal MI and unplanned repeat revascularization [23]. In addition, a study including 798 patients with T_2_DM and NSTE-ACS undergoing PCI reported that 1-unit increase of TyG index was independently associated with higher risk of primary endpoint (a composite of all-cause death, non-fatal MI and ischemia-driven revascularization)[HR: 3.208 per 1-unit increase, 95% Cl 2.40–4.29, *p* < 0.001], and the addition of TyG index to a baseline risk model had an incremental effect on the predictive value for adverse prognosis [AUC: baseline risk model, 0.800 vs. baseline risk model + TyG index, 0.856, *p* < 0.001] [25]. However, the predictive effects of the TyG index on MACCEs in patients with AMI combined with T_2_DM, are still unclear.

In this study, we investigated the prognostic value of the TyG index in patients with AMI combined with T_2_DM for the first time. To better understand the predictive power of TyG index for different CVEs, we analyzed the correlation between TyG index and each type of MACCEs (including all-cause death, CV death, non-fatal MI, non-fatal stroke, revascularization, and cardiac rehospitalization), which other studies have not tried. The conclusions drawn by this research have important guiding role for clinicians to predict the occurrence of future clinical events in patients with AMI and T_2_DM. In addition, we also found that adding TyG index to the baseline risk model had a significantly incremental effect on the predictive value for MACCEs, which is consistent with the conclusions of Zhao et al. [25]. Another novelty of this research is that we have done the predictive value of TyG index on MACCEs in different subgroups, including age, sex, BMI, smoker, HT, eGFR, LVEF and AMI type. We found that TyG index has a good predictive effect on MACCEs in most subgroups, except for patients with eGFR < 60 ml/min/1.73 m^2^. For this result, the mechanism is still unclear. There are relatively few studies on TyG index and kidney disease. Zhao et al. [32] found that an elevated TyG index was associated with a higher risk of nephric microvascular damage. Zhu et al. [33] showed that an elevated TyG index is signifcantly associated with HT in the subgroup of the lower eGFR(< 90 ml/min/1.73 m^2^). Maybe we need to do some work on that.

Our study had several limitations. First, this was a single-center study although including a large sample size; thus, generalization of the findings should be cautious. Second, laboratory parameters were only measured once after hospital admission, which could cause potential bias due to measurement error. Third, conventional laboratory testing methods for IR, such as HOMA-IR, has not been tested, the relationship between TyG index and IR cannot be verified directly in this study. In addition, prospective cohort studies are required to confirm our findings.

## Conclusions

In conclusion, the current study firstly demonstrated that elevated TyG index level was a strong independent predictor of MACCEs in patients with AMI and T_2_DM. In addition, adding the TyG index to a baseline risk model had an incremental effect on the predictive value for MACCEs.

## Data Availability

The datasets used and/or analyzed during the current study are available from the corresponding author on reasonable request.

## Abbreviations

TyG: Triglyceride-glucose
AMI: Acute myocardial infarction
T_2_DM: Type 2 diabetes mellitus
MACCEs: Major adverse cardiovascular and cerebrovascular events
IR: Insulin resistance
CV: Cardiovascular
CVDs: Cardiovascular diseases
CVEs: Cardiovascular events
TGs: Triglycerides
FPG: Fasting plasma glucose
HOMA-IR: The homeostasis model assessment of insulin resistance
CBD: Cardiovascular Center of Beijing Friendship Hospital Database
eGFR: Estimated glomerular filtration rate
CABG: Coronary artery bypass graft
SPISE: The Single Point Insulin Sensitivity Estimator
HDL-C: High-density lipoprotein cholesterol
BMI: Body mass index
RBG: Random blood glucose
OGTT: Oral glucose tolerance test
HT: Hypertension
TC: Total cholesterol
LDL-C: Low-density lipoprotein cholesterol
NSTEMI: Non-ST-segment elevation myocardial infarction
STEMI: ST-elevation myocardial infarction
ROC: Receiver operating characteristic
NRI: Net reclassification improvement
IDI: Integrated discrimination improvement
Hs-CRP: Hypersensitive C-reactive protein
PCI: Percutaneous coronary intervention
LVEF: Left ventricular ejection fraction
LM: Left main coronary artery
LAD: Left anterior descending
CKD: Chronic kidney disease
WBC: White blood cell
ACEI/ARB: Angiotensin-converting enzyme inhibitor/angiotensin receptor blocker
HR: Hazard ratio
HbA1c: Glycated hemoglobin
CAD: Coronary artery disease
MACEs: Major adverse cardiovascular events
NSTE-ACS: Non-ST-segment elevation acute coronary syndrome
